# Identification of cancer-type specific expression patterns for active aldehyde dehydrogenase (ALDH) isoforms in ALDEFLUOR assay

**DOI:** 10.1007/s10565-018-9444-y

**Published:** 2018-09-15

**Authors:** Lei Zhou, Dandan Sheng, Dong Wang, Wei Ma, Qiaodan Deng, Lu Deng, Suling Liu

**Affiliations:** 1 The CAS Key Laboratory of Innate Immunity and Chronic Disease, School of Life Sciences and Medical Center, University of Science & Technology of China, Hefei, 230027 Anhui China; 2 Shanghai Cancer Center & Institutes of Biomedical Sciences, Cancer Institute, Key Laboratory of Breast Cancer in Shanghai, Key Laboratory of Medical Epigenetics and Metabolism, Innovation Center for Cell Signaling Network, Shanghai Medical College, Fudan University, Shanghai, 200032 China; 3 Present Address: Key Laboratory of Breast Cancer in Shanghai, Cancer Institute, Fudan University Shanghai Cancer Center, Shanghai, 200032 China

**Keywords:** Aldehyde dehydrogenase (ALDH), ALDEFLUOR assay, Cancer stem cell (CSC), BAAA, DEAB

## Abstract

Aldehyde dehydrogenases (ALDHs) defend intracellular homeostasis by catalyzing the conversion of toxic aldehydes into non-toxic carboxylic acids, which is of particular importance to the self-renewal of stem cells and cancer stem cells. The widely used ALDEFLUOR assay was initially designed to indicate the activity of ALDH1A1 in leukemia and has been demonstrated to detect the enzyme activity of several other ALDH isoforms in various cancer types in recent years. However, it is still elusive which isoforms, among the 19 ALDH isoforms in human genome, are the potential contributors in catalyzing ALDEFLUOR assay in different cancers. In the current study, we performed a screening via overexpressing each ALDH isoform to assess their ability of catalyzing ALDEFLUOR assay. Our results demonstrate that nine isoforms are active in ALDEFLUOR assay, whose overexpression significantly increases ALDH-positive (ALDH^+^) population. Further analysis of the expression of these active isoforms in various cancers reveals cancer-type specific expression patterns, suggesting that different cancer types may exhibit ALDEFLUOR activity through expression of specific active ALDH isoforms. This study strongly indicates that a detailed elucidation of the functions for each active ALDH isoform in CSCs is necessary and important for a profound understanding of the underlying mechanisms of ALDH-associated stemness.

**Electronic supplementary material:**

The online version of this article (10.1007/s10565-018-9444-y) contains supplementary material, which is available to authorized users.

## Introduction

Tumor-initiating cell (TIC) or cancer stem cell (CSC) is a unique subpopulation of cancer cells endowed with stem cell-like capabilities of self-renewal, multi-potent differentiation, and infinite proliferation (Jordan et al. 2006; Shay and Wright 2010). The existence of CSCs has been widely accepted and regarded as the cause of cancer progression (Ayob and Ramasamy 2018), relapse, treatment resistance as well as metastasis (Chang 2016). Among the numerous CSC markers, such as CD24^−^/CD44^+^ (Al-Hajj et al. 2003) and CD133^+^ (Miraglia et al. 1997), ALDH is quite unique as it detects endogenous enzyme activity to characterize the “stemness” of CSCs rather than cell surface molecules, which is more explicitly related to stemness as this activity is critical for the self-renewal of stem cells. After its initial applications in leukemia (Storms et al. 1999) and breast cancer (Ginestier et al. 2007), ALDH^+^ population has been applied to identifying CSCs in many other cancer types, such as lung cancer (Sullivan et al. 2010), pancreatic cancer (Kim et al. 2011), colorectal cancer (Shenoy et al. 2012), and prostate cancer (van den Hoogen et al. 2010). Combination of ALDH^+^ with other CSC markers enriches CSCs with better specificity for certain cancer types (Ginestier et al. 2007; Silva et al. 2011), reflected by higher CSC frequency of the enriched cells compared to single CSC marker.

The method for detecting ALDH enzyme activity in CSCs is the ALDEFLUOR assay, in which the cells with ALDH enzyme activity are able to convert a fluorescent aldehyde (BODIPY-aminoacetaldehyde, BAAA) to its corresponding carboxylic acid (BODIPY-aminoacetate, BAA) (Storms et al. 1999). The product BAA is negatively charged in the circumstance of cytoplasm and is retained inside the cells, the accumulation of which can be detected and analyzed by flow cytometry. Initially, ALDEFLUOR assay was designed to determine the activity of ALDH1A1 (Jones et al. 1995; Storms et al. 1999), which had been previously demonstrated to be expressed in TICs of leukemia at a higher level than non-TICs (Kastan et al. 1990). However, as we know, there are 19 ALDH isoforms identified in human genome so far (Vasiliou and Nebert 2005) and some of these isoforms, besides ALDH1A1, have been shown to exhibit activity in ALDEFLUOR assay, namely, ALDH1A2 (Moreb et al. 2012), ALDH1A3 (Marcato et al. 2011b), and ALDH2 (Garaycoechea et al. 2012; Moreb et al. 2012). Therefore, it is reasonable to propose that other ALDH isoforms may be potential contributors in ALDEFLUOR assay and hence play a role in CSC regulation (Marcato et al. 2011a).

In addition, although ALDH1A1 has long been regarded as a marker for poor prognosis in several cancer types and the ALDEFLUOR assay defined ALDH^+^ portion has long been owed to ALDH1A1, several groups have analyzed the expression of ALDH isoforms in proteomic data (Zhang et al. 2011) or transcriptomic data (Chang et al. 2018) of several cancer types and unraveled that some other ALDH isoforms are either positively or negatively correlated to prognosis. These results indicate that many other ALDH isoforms could play a role in cancer suppression or progression in a cancer-type dependent manner. Not surprisingly, several ALDH isoforms other than ALDH1A1 have been proved being associated with certain cancers, e.g., ALDH3A1 in lung cancer (Patel et al. 2008) and prostate cancer (Yan et al. 2014) and ALDH5A1 in breast cancer (Kaur et al. 2012). On the other side, ALDH1A2 (Kim et al. 2005) and ALDH2 (Jin et al. 2015) have been reported to act as tumor suppressors in prostate cancer and liver cancer, respectively. These results remind us that a portion of the so-called ALDH^+^ cancer stem cells identified by ALDEFLUOR assay may be not cancer stem cells at all, if these cells are lit up by unknown tumor suppressor ALDH isoforms with ALDEFLUOR activity. For this reason, identification of active ALDH isoforms in ALDEFLUOR assay and discrimination of their roles in cancer progression are more urgent in some sense.

To shed light on the elusive question about contribution of ALDH isoforms in ALDEFLUOR assay, we performed a screening of all 19 ALDH isoforms to determine whether they exhibit activities in ALDEFLUOR assay by overexpression with lentiviral vectors. Intriguingly, our abovementioned hypothesis is confirmed by the results, revealing that nine out of 19 ALDH isoforms are able to catalyze ALDELFUOR assay. Several of these isoforms have been reported by other groups to be involved in ALDEFLUOR assay, namely ALDH1A1 (Jones et al. 1995), ALDH1A2 (Moreb et al. 2012), ALDH1A3 (Marcato et al. 2011b), and ALDH2 (Garaycoechea et al. 2012; Moreb et al. 2012), while the other five ones have seldom been described and their role in cancer have not yet been explored in detail.

In summary, in this study, we have identified nine ALDH isoforms that are contributors in ALDEFLUOR assay in a cancer-type specific manner. When overexpressed, these isoforms remarkably increased the ALDH^+^ cells, whereas other isoforms did not show similar effects. In addition, mutation in the activity center of these isoforms obviously abolished this effect, indicating that the elevated ALDH^+^ proportion is due to the activity of these ALDH isoforms themselves, but not due to altered activity of other ALDH enzymes in the cells. Our results definitely answer a long-standing question, that is, which of the 19 ALDH isoforms in human genome are contributors to ALDEFLUOR assay in CSC identification. Hence, our work would be of great value in the field of CSC research to identify CSCs with ALDEFLUOR assay.

## Materials and methods

### Plasmids and cDNAs

The lentiviral vectors pSIN-EF-FLAG-EcoRI-pur (pSIN-FLAG-E) and pSIN-EF-BamHI-FLAG-pur (pSIN-B-FLAG) were kind gifts from Professor Mian Wu (University of Science and Technology of China), which contain appropriate restriction endonuclease sites for molecular cloning and a FLAG tag for subsequent detection of the expressed proteins. The cDNAs of ALDH1A2, ALDH1B1, ALDH3A1, ALDH3A2, ALDH4A1, ALDH5A1, ALDH16A1, and ALDH18A1 were kindly provided by Professor Jiahuai Han (Xiamen University, China). The cDNAs of ALDH1L1, ALDH1L2, ALDH3B2, and ALDH8A1 were synthesized at Synbio Technologies (Suzhou, China). The cDNAs for other ALDH isoforms were amplified by PCR from reverse-transcribed cell line cDNA samples kept in our laboratory. In detail, ALDH1A3, ALDH3B1, and ALDH7A1 were cloned from SUM149; ALDH2, ALDH6A1, and ALDH9A1 from SK-Br-3; ALDH1A1 from BT474.

### Plasmid construction

In order to construct overexpressing vectors, we amplified the coding sequences by PCR and cloned them into either pSIN-FLAG-E or pSIN-B-FLAG vector with N- or C-terminus fused FLAG tag, respectively, in order not to disrupt its endogenous localization. The N-terminal fusion isoforms are listed as follows: ALDH1A1, ALDH1A2, ALDH1A3, ALDH3A1, ALDH3A2, ALDH3B1, ALDH3B2, ALDH5A1, ALDH7A1, ALDH8A1, ALDH9A1, and ALDH16A1. The remaining isoforms, including ALDH1B1, ALDH1L1, ALDH1L2, ALDH2, ALDH4A1, ALDH6A1, and ALDH18A1, were cloned with FLAG fused to their C termini. Primers used to clone the overexpressing vectors are listed in supplementary Table S1. The PCR-amplified DNA fragments were cloned into the digested vector via seamless cloning kit (C112–02, Vazyme) following the manufacturer’s recommendations. The high fidelity DNA polymerase PrimeStar MasterMix (#R045A) used in this work was purchased from Takara. Mutation of activity sites of ALDH^a^ isoforms was completed using overlapping PCR; the primers used were listed in Supplementary Table S2. The shRNA vectors used in this study to interfere ALDH1A1, ALDH1A3, ALDH2, ALDH3A2, and ALDH3B1 were constructed with primers listed in Supplementary Table S4. All plasmids constructed in this work were sequenced to ensure that the correct sequences were cloned.

### Lentiviral production and infection

The ALDH isoform-overexpressing vectors were co-transfected with plasmids encoding gag/pol and VSVG (2 μg:1 μg:1μg) into packaging cells using 12 μg Polyethylenimine (765090-1G, Sigma) and incubated for 48 h before harvesting of the supernatant. HEK293T, SUM159, and MDA-MB-231 cells were infected with the lentivirus-containing supernatant in the presence of polybrene (107689-10G, Sigma) for 24 h and selected with 5 μg/mL puromycin (Gibco) for at least 7 days to establish stable cell lines.

### Cell culture

HEK293T and MDA-MB-231 cells were purchased from ATCC and cultured according to the ATCC protocols, with HEK293T maintained in high-glucose DMEM (Gibco, USA) supplemented with 10% FBS (Gibco, USA) and 1% pen-strep antibiotic (Beyotime, China) and MDA-MB-231 in RPMI1640 medium (Gibco, USA) supplemented with 10% FBS (Gibco, USA) and 1% pen-strep antibiotic (Beyotime, China). SUM159 cell was purchased from Asterland (Neve et al. 2006) and cultured with Han’s F-12 (Invitrogen) supplemented with 5% FBS (Gibco, USA), 5 μg/mL insulin, and 1 μg/mL hydrocortisone, 4 μg/mL gentamicin (Sigma, USA) and 1% pen-strep (Beyotime, China). All cell lines were maintained at 37 °C in an atmosphere of 5% CO2. Mycoplasma contamination was routinely detected to avoid unexpected interference to the scientific results.

### ALDEFLUOR assay and flow cytometry

ALDEFLUOR assay kit was purchased from Stem Cell Technology (#01700) and was performed following the manufacturer’s protocol with only a few modifications. In brief, 1 million cells were resuspended in 1 ml ALDEFLUOR buffer. After addition of 5 μL BAAA and a brief mixing, 300 μL of the cell suspension was immediately transferred to another tube supplemented with 5 μL DEAB and pipetted to mix evenly. Both tubes were then placed into cell incubator to allow the reaction to occur at 37 °C for 40 min. Before analyzing by flow cytometry, the cells were washed twice with 2 ml ALDEFLUOR buffer and eventually resuspended in 500 μL ALDEFLUOR buffer supplemented with DAPI to stain for dead cells. Analysis of the samples were completed on Moflo Astrios or CytoFLEX (Beckman Coultier), equipped with a 405/448 channel to delineate dead cells and a 488/513 channel to collect the signal of the fluorescent dye (BAAA and BAA) used in ALDEFLUOR assay.

### Quantitative real-time PCR

Total RNA was extracted by Trizol (#9109, Takara) and cDNA was generated by reverse transcription (Q111-02, Vazyme) according to the manufacturers’ recommendations. Quantitative real-time PCR (qRT-PCR) was performed using a SYBR Green Kit (R211-02, Vazyme, China) and an ABI-7500 device (Applied Bioscience, USA). Each sample was normalized to GAPDH and amplification products were tested for specificity by melting curves. Quantitative PCR primers are listed in Supplementary Table S3.

### Western blotting

Cells were harvested by scraping in the presence of RIPA lysis buffer after PBS washing. After lysing for 30 min on ice, the cell debris was removed by centrifuge at 12000*g* for 10 min. Protein concentration of the supernatant was determined using the BCA kit (Thermo Scientific). After being boiled with loading buffer, the samples were separated in 10% SDS-PAGE gel and transferred to PVDF membrane (Millipore). Blocked in 5% nonfat milk for 1 h, the membrane was incubated with primary antibody overnight at 4 °C. Primary antibodies anti-GAPDH (TransGen; HC301-01, 1:1000), anti-FLAG (Sigma; F7425, 1:2000) and subsequent second antibodies Anti-Mouse (TransGen; HS201-01, 1:5000), Anti-Rabbit (TransGen; HS101-01, 1:5000) were used to detect the specific proteins.

### Immunofluorescence staining and confocal imaging

SUM159 cells were plated in slide chambers (#154526, Thermo Scientific) and cultured for 24 h to attach. After twice washing with PBS, cells were fixed with 4% PFA (paraformaldehyde) (E672002-0500, Sangon Biotech) at room temperature for 15 min, followed by membrane permeabilizing with 0.2% Triton X-100 (TB0198, Sangon Biotech) for 5 min and blocking with 1% BSA for 30 min at room temperature. Primary antibody against FLAG (Sigma; F7425, 1:200) was incubated at 4 °C overnight, followed by fluorescence-conjugated secondary antibody (A11035, Life Technologies, 1:500) incubation at room temperature for 1 h. Cell nuclei were stained with DAPI (P36931, Life Technologies). Slides were mounted following twice washing with PBS. Images were captured with confocal microscope (TCS SP5 II, Leica) with × 63 oil objective lens.

## Results

As there are 19 ALDH isoforms identified in human genome and some of them have been reported to play a role in specific cancer types, we wonder if the expression of these isoforms exhibits a cancer-type specific pattern in different cancers. To this end, we retrieved and analyzed the RNA-chip data from CCLE (Barretina et al. 2012) of cell lines for various cancers. We chose several solid cancer types that have been reported to contain ALDH^+^ CSCs, including breast (Ginestier et al. 2007), lung (Sullivan et al. 2010), ovary (Silva et al. 2011), liver (Ma et al. 2008), skin (Luo et al. 2013), kidney (Yuan et al. 2016), pancreas (Kim et al. 2011), and esophagus (Zhang et al. 2012). Our results indeed indicate a cancer-type specific expression pattern of these 19 ALDH isoforms (Supplementary Fig. S1), demonstrated by the observation that different cancer types show a preferential expression of certain isoforms. For instances, a large part of breast cancer cells shows higher level of ALDH1A3, consistent with a previous report (Marcato et al. 2011b) claiming ALDH1A3 to be the main contributor in ALDEFLUOR assay in breast cancer, whereas liver cancer and kidney cancer show high level of ALDH1A1. The cancer-type specific expression patterns imply that different cancers may utilize specific ALDH isoforms or combinations to show ALDH activity, making it more urgent to identify the active ALDH isoforms contributing the enzymatic activity in ALDEFLUOR assay.

**Fig. 1:**
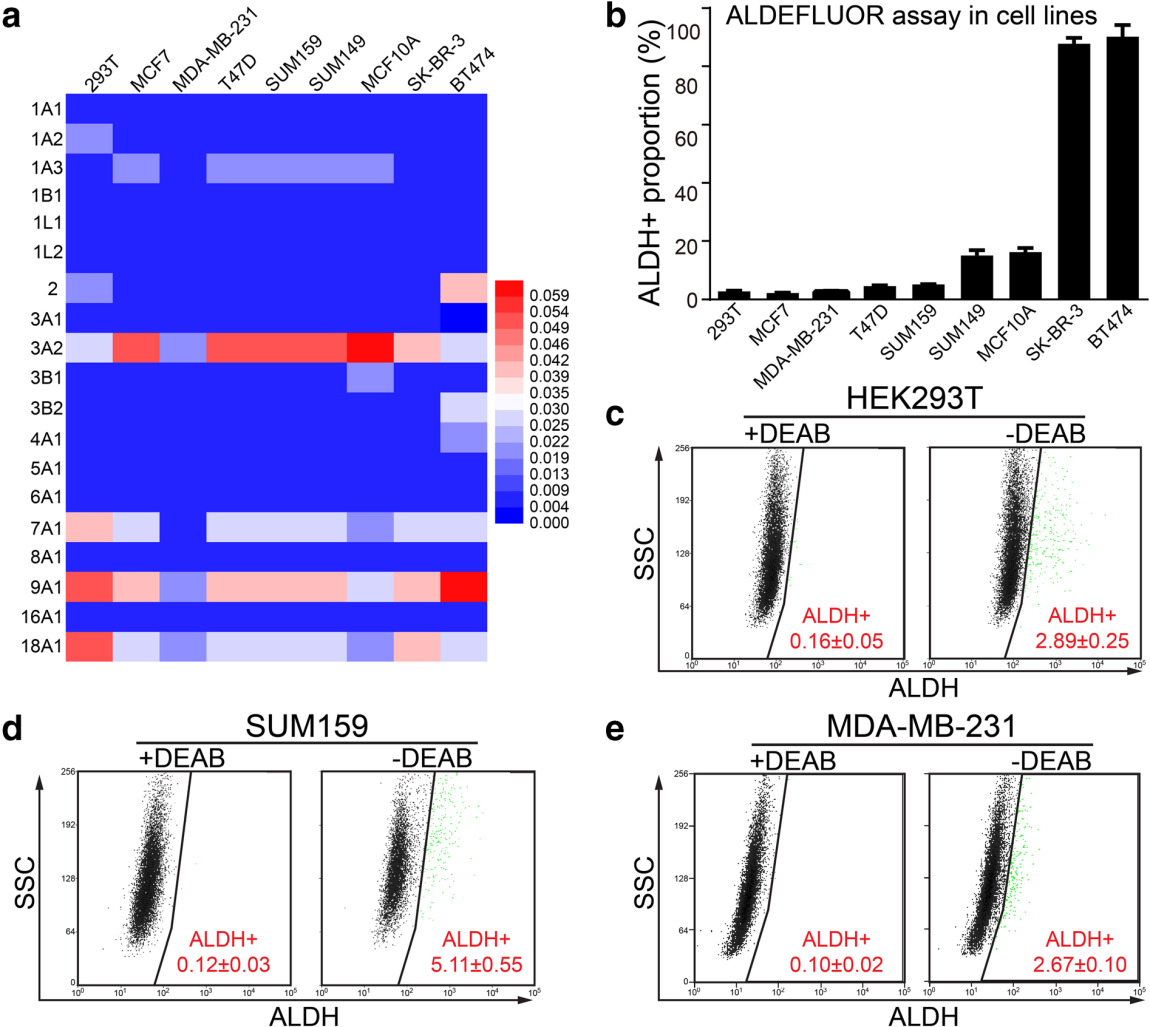
Determination of ALDH isoform expression and the background ALDH^+^ proportion in cell lines. **a** qRT-PCR was performed to determine the endogenous level of ALDH isoforms in several cell lines with GAPDH as loading control. Based on the relative expression value of qRT-PCR, heatmap was generated by HemI (Deng et al. 2014), with color code of expression value shown on the right side. **b**–**e** ALDEFLUOR assay was performed on indicated parental cell lines to determine the background ALDH^+^ proportion. Representative images were shown for HEK293T, SUM159, and MDA-MB-231. Triple experiments were carried and the ALDH^+^ percentage is expressed as mean ± SEM. DEAB was routinely used to provide a negative control in order to set a threshold. Flow cytometry data was illustrated as ALDH activity (ALDH) vs. side scatter signal (SSC)

To identify the ALDH isoforms that are potentially active in ALDEFLUOR assay, we cloned all 19 ALDH isoforms into lentiviral vectors and then established stable overexpression cell lines of HEK293T, SUM159, and MDA-MB-231. We chose these three cell lines because they exhibit relatively low endogenous level of most ALDH isoforms (Fig. 1a), and they also show relatively low background ALDH^+^ proportion in ALDEFLUOR assay (Fig. 1b–e), which are conducive to defining the changes in ALDEFLUOR activity after overexpression of ALDH isoforms.

**Fig. 2:**
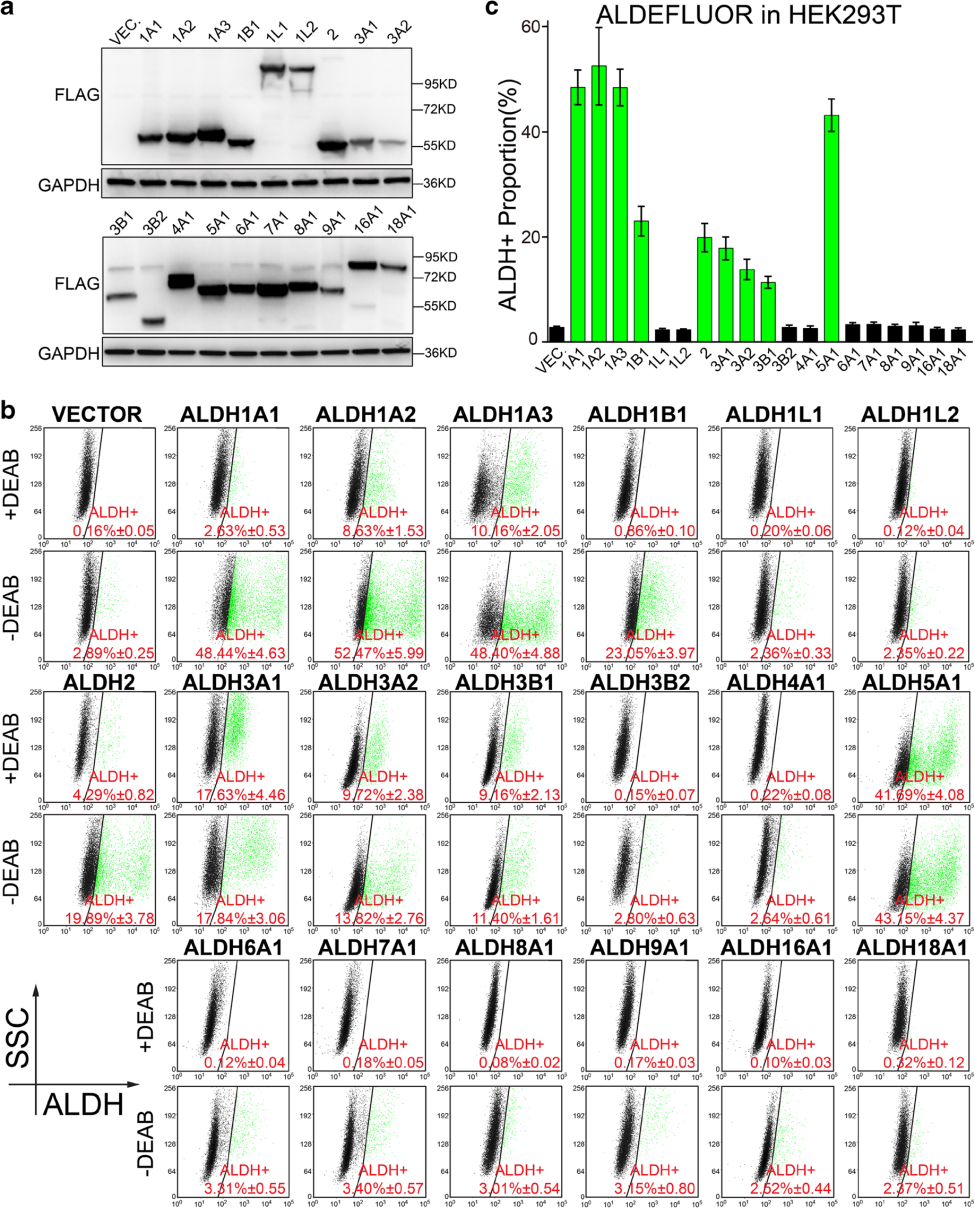
Nine ALDH isoforms are identified to be active in ALDEFLUOR assay in HEK293T cell line. **a** Western blotting was performed to confirm the overexpression of ALDH isoforms in HEK293T cells, with GAPDH used as loading control. Molecular weight for marker proteins is indicated on the right side. **b**, **c** ALDEFLUOR assay was performed to determine the ALDH^+^ proportion in HEK293T cells overexpressing ALDH isoforms. Representative pictures are shown in (**b**) and the statistical values of ALDH^+^ percentage are shown in (**c**). Triple independent experiments were performed. Percentage of ALDH^+^ cells is shown as mean ± SEM

**Fig. 3:**
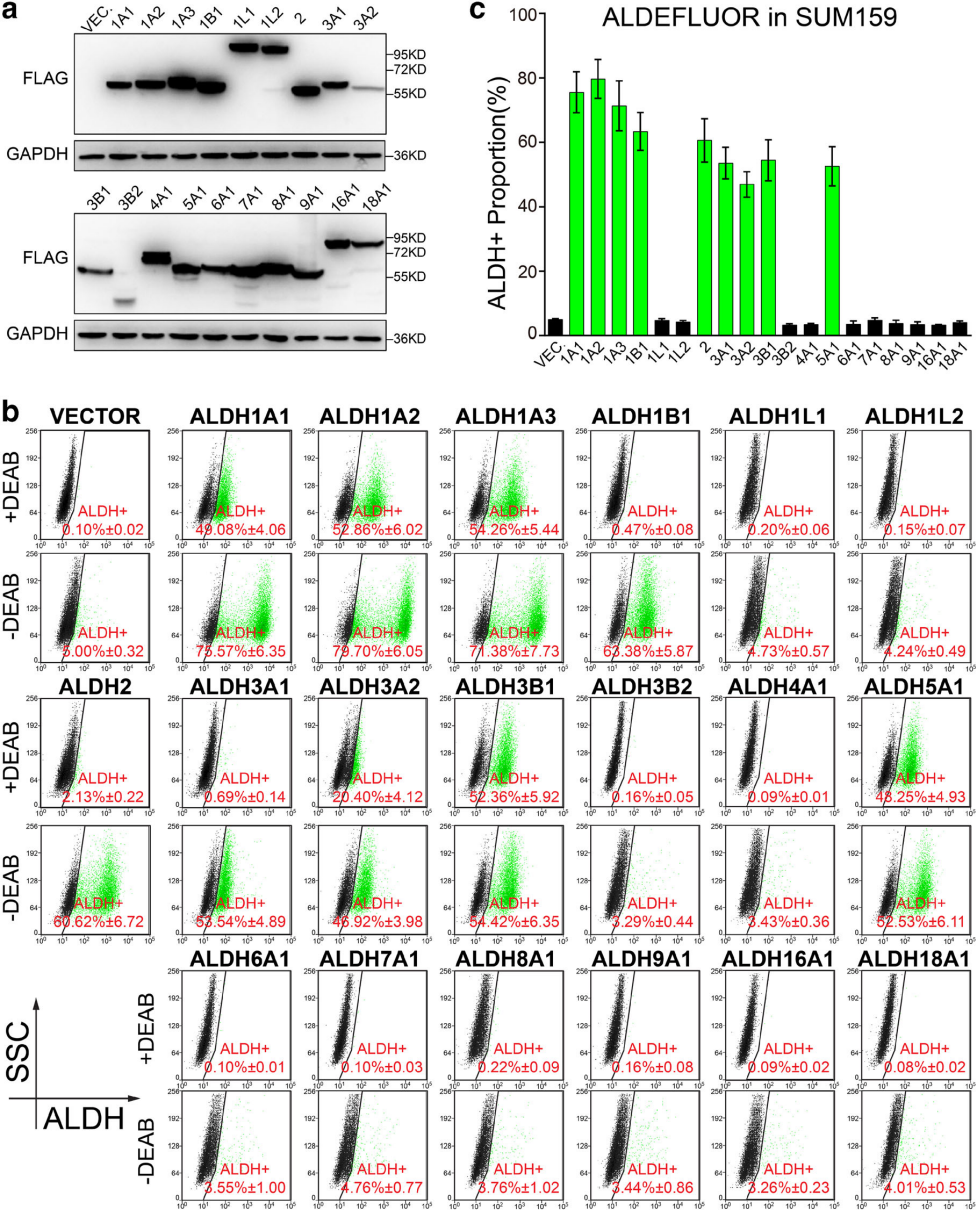
Nine ALDH isoforms are identified to be active in ALDEFLUOR assay in SUM159 cell line. **a** Western blotting was performed to confirm the overexpression of ALDH isoforms in SUM159 cells, with GAPDH used as loading control. Molecular weight for marker proteins is indicated on the right side. **b**, **c** ALDEFLUOR assay was performed to determine the ALDH^+^ proportion in SUM159 cells overexpressing ALDH isoforms. Representative pictures are shown in (**b**) and the statistical values of ALDH^+^ percentage are shown in (**c**). Triple independent experiments were performed. Percentage of ALDH^+^ cells is shown as mean ± SEM

**Fig. 4:**
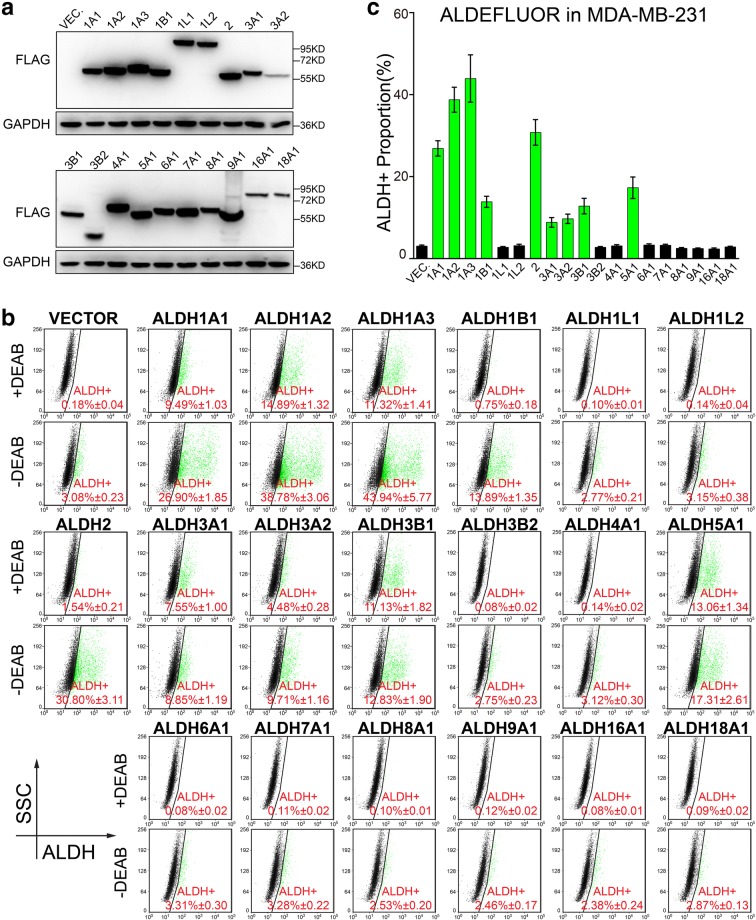
Nine ALDH isoforms are identified to be active in ALDEFLUOR assay in MDA-MB-231 cell line. **a** Western blotting was performed to confirm the overexpression of ALDH isoforms in MDA-MB-231 cells, with GAPDH used as loading control. Molecular weight for marker proteins is indicated on the right side. **b**, **c** ALDEFLUOR assay was performed to determine the ALDH^+^ proportion in MDA-MB-231 cells overexpressing ALDH isoforms. Representative pictures are shown in (**b**) and the statistical values of ALDH^+^ percentage are shown in (**c**). Triple independent experiments were performed. Percentage of ALDH^+^ cells is shown as mean ± SEM

Before detecting their activity in ALDEFLUOR assay, we firstly confirmed the overexpression of these ALDH isoforms by Western blotting (Figs. 2a, 3a and 4a), and our results demonstrated that all ALDH isoforms are successfully over-expressed in all three cell lines. We then performed ALDEFLUOR assay and determined the ALDH^+^ proportion. Interestingly, our results indicate that nine out of the 19 ALDH isoforms are potentially involved in ALDEFLUOR assay, as overexpression of these isoforms result in significantly higher ALDH^+^ proportion than the control groups (Figs. 2b–c, 3b–c and 4b–c). For abridged notation, we name this subset of ALDH isoforms as active ALDH (ALDH^a^) isoforms, including ALDH1A1, ALDH1A2, ALDH1A3, ALDH1B1, ALDH2, ALDH3A1, ALDH3A2, ALDH3B1, and ALDH5A1, and name the others as non-active ALDH (ALDH^n^).

Our results also imply that the ALDH inhibitor (DEAB) used in ALDEFLUOR assay, is not specific to ALDH1A1 (Figs. 2b, 3b and 4b). In fact, DEAB inhibits a series of ALDH^a^ isoforms to different extents except for ALDH3B1 and ALDH5A1, which are not significantly inhibited by DEAB. Our result is, in part, consistent to a previous study, where C. A. Morgan et al. demonstrated in vitro assays that DEAB is an inhibitor to various ALDH isoforms (Morgan et al. 2015). However, this study did not cover ALDH3A2 and ALDH3B1, which are shown in our study to exhibit ALDH activity in ALDEFLUOR assay and are merely inhibited by DEAB.

**Fig. 5:**
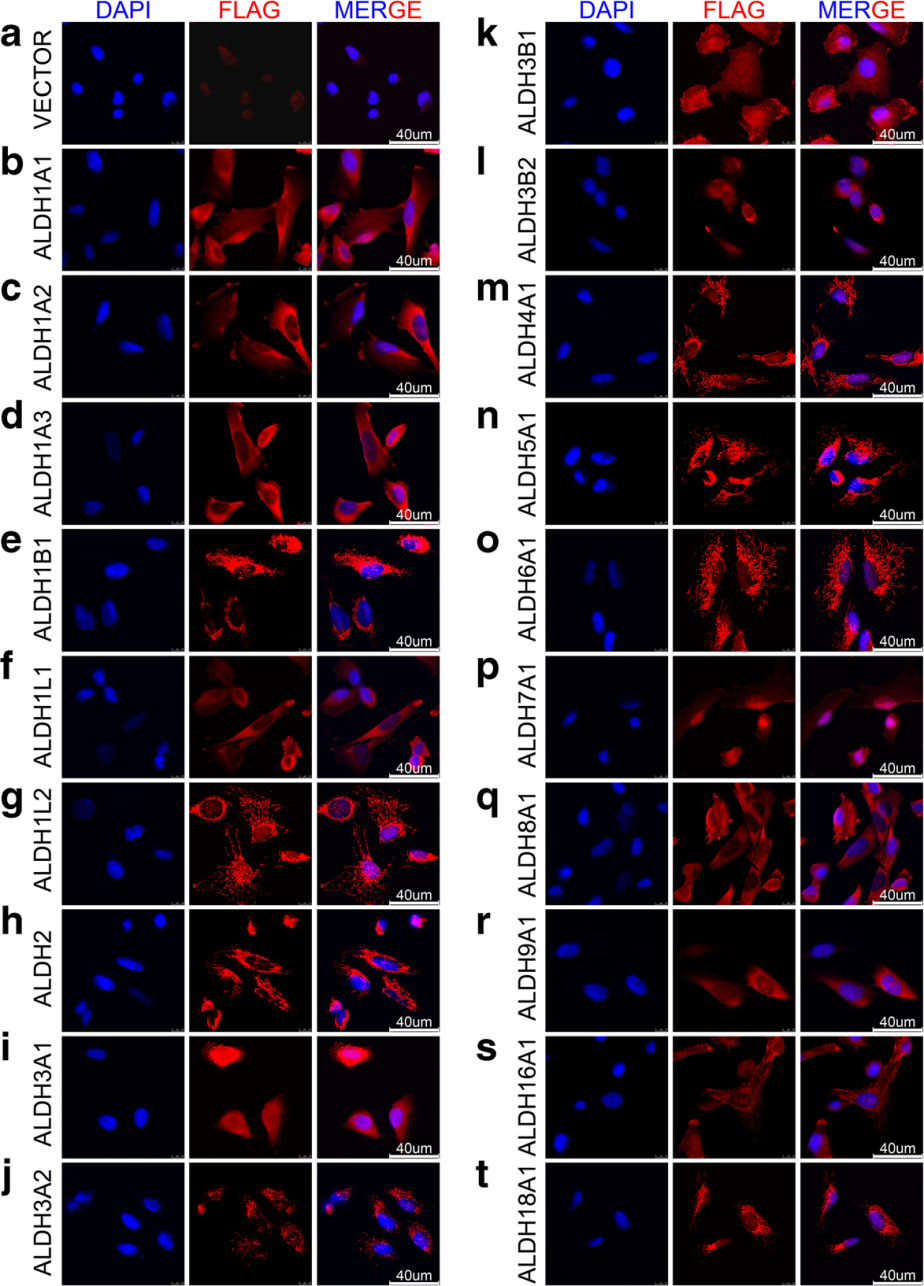
Localization of the FLAG-tagged ALDH isoforms was confirmed by immunofluorescence in SUM159 cells. **a** Vector. **b** ALDH1A1 (cytosol and nucleus) (Kahlert et al. 2012). **c** ALDH1A2 (cytosol) (Marchitti et al. 2008). **d** ALDH1A3 (cytosol) (Grun et al. 2000). **e** ALDH1B1 (mitochondrion) (Stagos et al. 2010a). **f** ALDH1L1 (cytosol) (Kang et al. 2016). **g** ALDH1L2 (mitochondrion) (Krupenko et al. 2010). **h** ALDH2 (mitochondrion) (Jin et al. 2015). **i** ALDH3A1 (cytosol and nucleus) (Stagos et al. 2010b). **j** ALDH3A2 (peroxisome) (Ashibe et al. 2007). **k** ALDH3B1 (membrane) (Kitamura et al. 2013). **l** ALDH3B2 (peri-nucleus) (Kitamura et al. 2015). **m** ALDH4A1 (mitochondrion) (Yoon et al. 2004). **n** ALDH5A1 (mitochondrion) (Hearl and Churchich 1984). **o** ALDH6A1 (mitochondrion) (Kedishvili et al. 1992). **p** ALDH7A1 (nucleus and cytosol) (Brocker et al. 2010). **q** ALDH8A1 (cytosol) (Lin and Napoli 2000). **r** ALDH9A1 (cytosol) (Lin et al. 1996). **s** ALDH16A1 (cytosol and membrane) (Vasiliou et al. 2013). **t** ALDH18A1 (mitochondrion) (Panza et al. 2016). The detected localization is indicated in parentheses, followed by supporting references. DAPI is used to counter-stain the nucleus. Bar (40 um) is shown at the bottom-right corner in the merged images

**Fig. 6:**
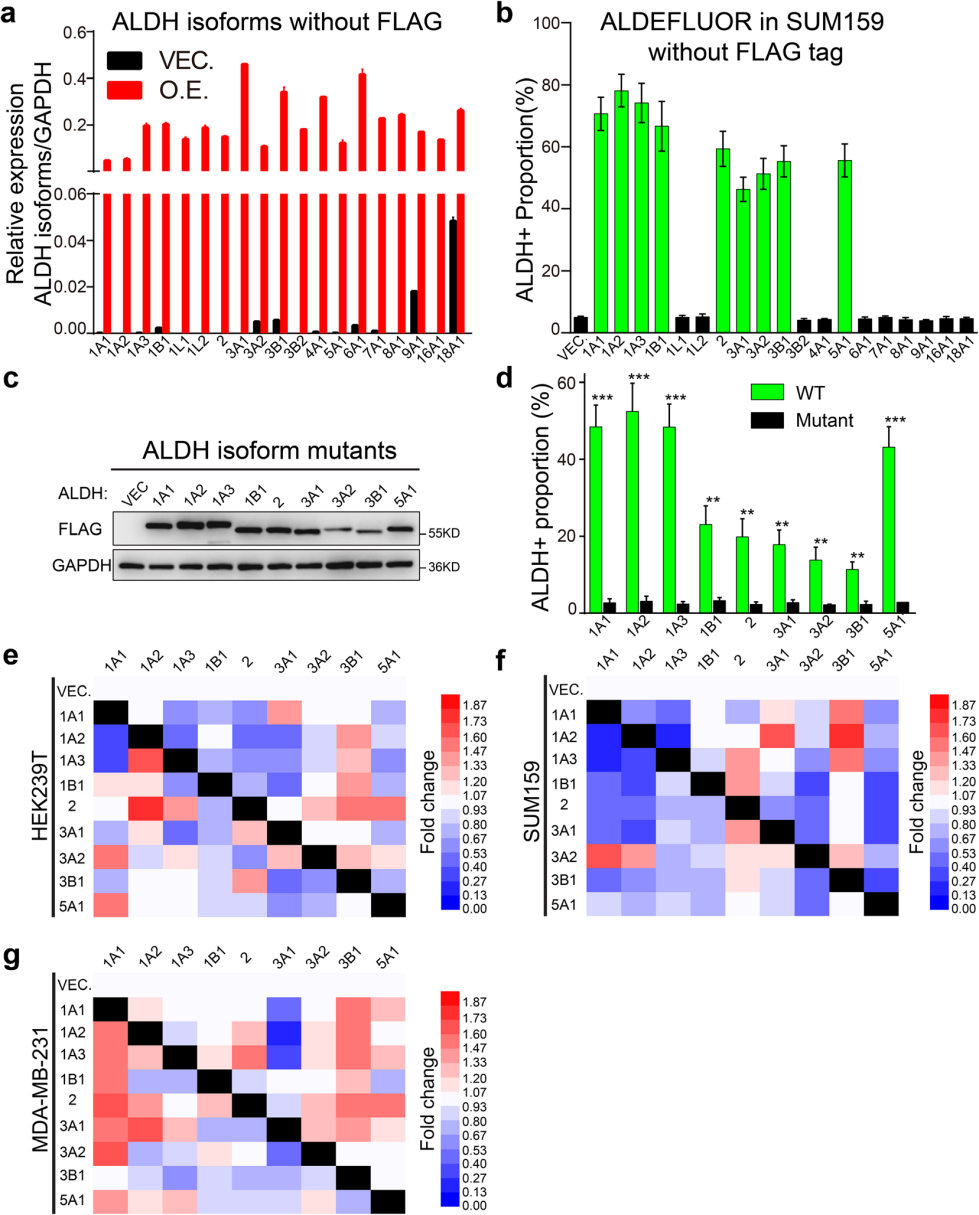
Expression of active ALDH^a^ isoforms is essential for cells to exhibit ALDH activity in ALDEFLUOR assay. **a** Quantitative real-time PCR was performed to confirm the overexpression of ALDH isoforms without FLAG tag in SUM159, with GAPDH as internal control. **b** ALDEFLUOR assay was performed to determine the activity of the ALDH isoforms without FALG tags in SUM159. Data are shown as mean ± SEM; *n* = 3 independent experiments. **c** The activity center of the nine ALDH^a^ isoforms was mutated and stable overexpressing HEK293T cell lines were established. Overexpression was confirmed by Western blotting, with GAPDH as loading control and the molecular weight for marker proteins is indicated on the right side. **d** ALDH activity was determined for the ALDH^a^ isoform mutants in ALDEFLUOR assay. Data are shown as mean ± SEM; *n* = 3 independent experiments, two-tailed Student’s *t* test, **p < 0.01 ***p < 0.001. **e**–**g** Expression of other ALDH^a^ isoforms was determined by qRT-PCR in HEK293T, SUM159, and MDA-MB-231, with GAPDH as internal control. The fold change was calculated according to the relative expression value of the empty vector (VEC.) group. Based on the fold change value, heatmap was generated by HemI (Deng et al. 2014)

To eliminate the worry about the use of FLAG tag, which may disturb the localization or the structure of the tagged proteins, Immunofluorescence staining of the FLAG-tagged ALDH proteins was performed to confirm the localization of the exogenously introduced proteins in SUM159 (Fig. 5) and the results are consistent with previous studies (Marchitti et al. 2008) and the Human Protein Atlas project (Thul and Lindskog 2018). We also constructed overexpressing plasmids without FLAG tag and established stable overexpressing cell lines of SUM159 (Fig. 6a). ALDEFLUOR assay of these overexpressing cells showed similar results to that of the FLAG-tagged group (Figs. 3c and 6b), demonstrating that our FLAG-tagged strategy is not likely to impair the activity, in particular, of the ALDH^n^ isoforms.

**Fig. 7:**
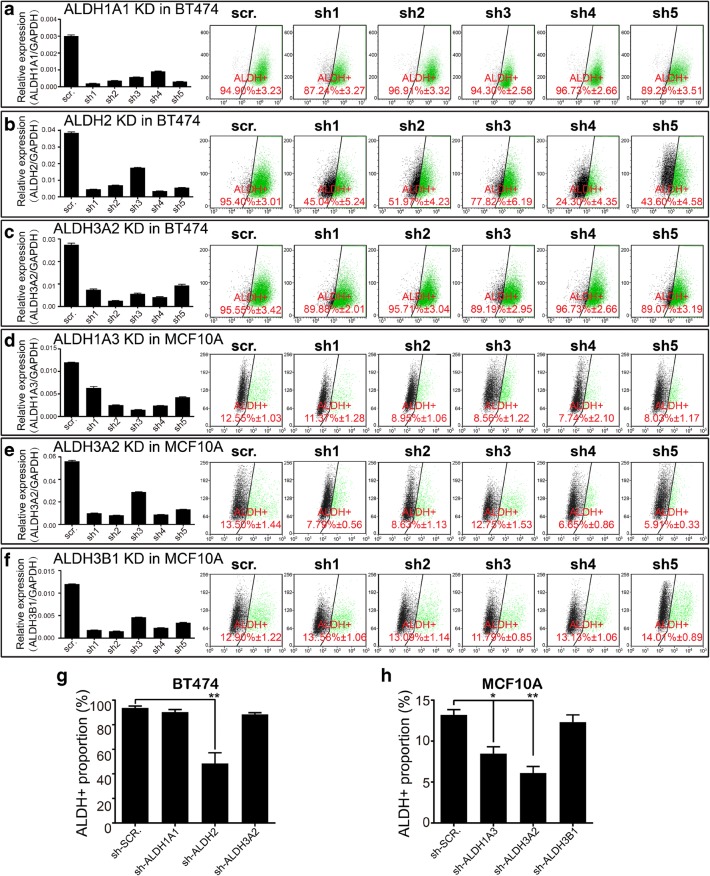
Interference of major endogenous ALDH^a^ isoforms in cell lines decreased ALDH^+^ percentage. **a**–**f** Major ALDH isoforms were stably knocked down by shRNA, and ALDEFLUOR assay was performed to determine the ALDH activity after knockdown of selected ALDH isoforms in BT474 and MCF10A, in which the selected ALDH isoforms are expressed at relatively high level. The knockdown efficiency of shRNAs were determined by qRT-PCR, data shown as mean ± SEM; *n* = 3 independent experiments. ALDEFLUOR assay was performed to determine the ALDH^+^ percentage after knockdown of specific ALDH isoforms. ALDH^+^ percentage is shown as mean ± SEM; *n* = 3 independent experiments. **g**, **h** Statistical data are shown for BT474 and MCF10A with ALDH isoforms knocked down. The values of efficient shRNAs in (**a**–**f)** are included only. Data are shown as mean ± SEM; two-tailed Student’s *t* test; *p < 0.05; **p < 0.01. shRNA, short hairpin RNA

However, it is also possible that the elevated ALDH^+^ population was a consequence of ALDH^a^ overexpression-induced signaling pathway alterations, which may be associated with increased endogenous ALDH activity. To solve this, on the one hand, we mutated the activity center of the ALDH^a^ isoforms and established stable overexpressing cell lines (Fig. 6c), which is expected to abolish the increased ALDH^+^ proportion if these isoforms are the direct contributors. As expected, mutations in the activity center of all nine ALDH^a^ isoforms obviously eliminated the increased ALDH^+^ proportion (Fig. 6d). On the other hand, we determined the endogenous expression level of all other ALDH^a^ isoforms upon overexpression of each ALDH^a^ isoform in all three tested cell lines and found no significant and coincident upregulation of other ALDH^a^ isoforms (Fig. 6e–g). These results provide evidence that the activity of the nine ALDH^a^ isoforms in ALDEFLUOR assay is not a result of the altered cell signaling network or induction of endogenous active ALDH isoforms but a direct consequence of the activity of these ALDH^a^ isoforms themselves. Further, to demonstrate that the ALDH^a^ isoforms defined by us may be responsible to ALDEFLUOR assay at endogenous level, we stably knocked down major ALDH^a^ isoforms (top three of the nine) in BT474 and MCF10A (Fig. 1a and 7a–f). As shown by our results, knockdown of ALDH2 in BT474 as well as ALDH1A3 and ALDH3A2 in MCF10A significantly decreased the ALDH^+^ proportion (Fig. 7g–h). These results suggest that ALDH2 is the major player in ALDEFLUOR assay of BT474, while ALDH1A3 and ALDH3A2 play important roles in ALDEFLUOR assay of MCF10A.

**Fig. 8:**
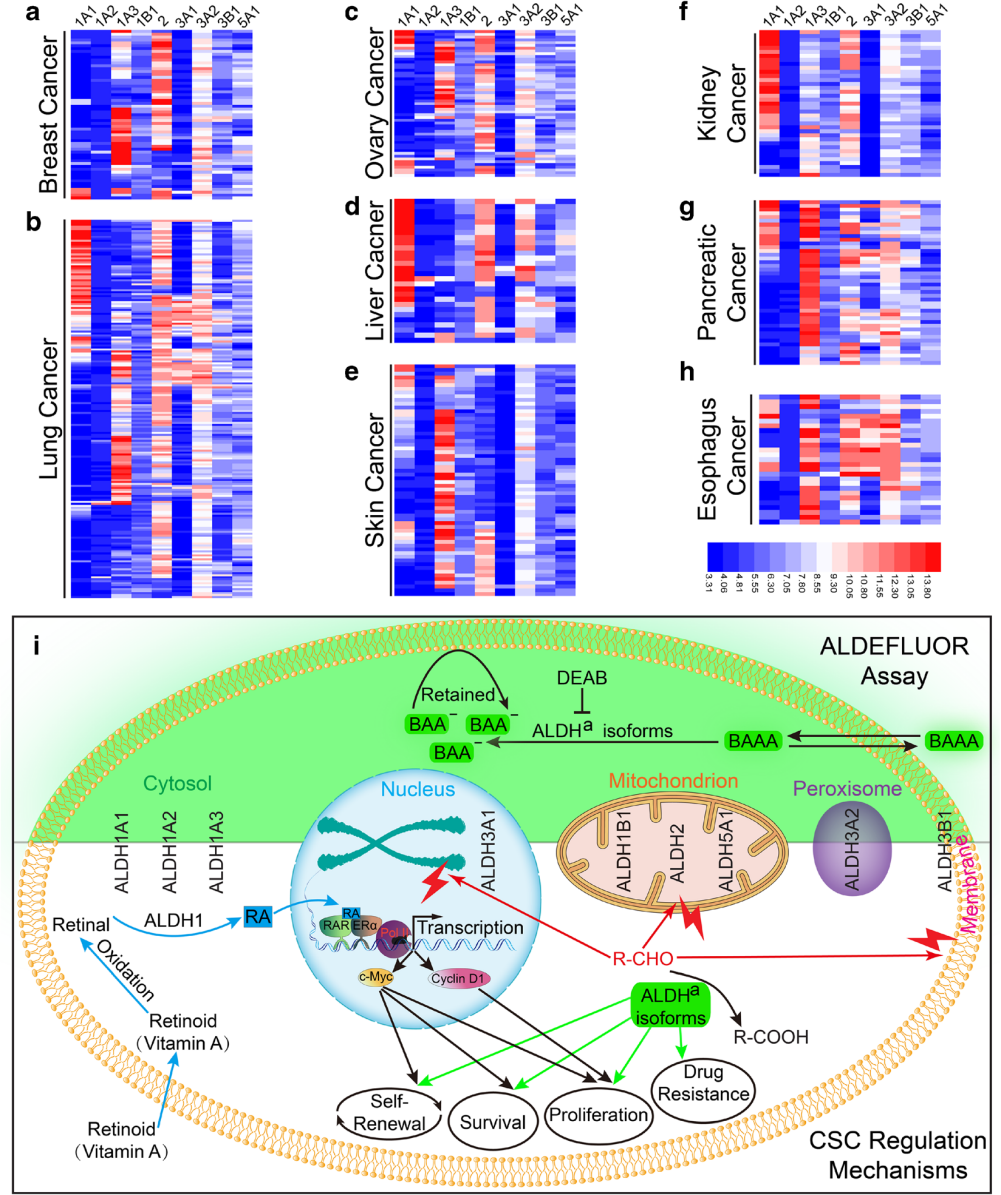
Different types of cancer exhibit unique expression pattern of ALDH^a^ isoforms. **a**–**h** RNA expression data for ALDH^a^ isoforms (ALDH1A1, ALDH1A2, ALDH1A3, ALDH1B1, ALDH2, ALDH3A1, ALDH3A2, ALDH3B1, and ALDH5A1) was collected from CCLE and illustrated as heatmap by the HemI tool (Deng et al. 2014) with hierarchical average linkage. Data was retrieved and analyzed for breast cancer (**a**), lung cancer (**b**), ovary cancer (**c**), liver cancer (**d**), skin cancer (**e**), kidney cancer (**f**), pancreatic cancer (**g**), and esophagus cancer (**h**). **i** A summary graph is illustrated to show the enzymatic function of ALDH^a^ isoforms in ALDEFLUOR assay and their possible roles in CSC regulation. ALDH^a^ isoforms are shown according to their major subcellular localizations. In the upper panel (light green area), the role of ALDH^a^ isoforms in ALDEFLUOR assay is illustrated, where the fluorescent BAAA is converted by ALDH^a^ isoforms to its negatively charged product BAA^−^ and retained in the cell. The lower panel (white) shows that the ALDH^a^ isoforms are involved in CSC regulation either via its detoxifying function of aldehydes, which is detrimental to cellular components, such as DNA, mitochondrion, and membrane (red flashes) or through the production of retinoic acid (RA) by ALDH1 family (ALDH1A1, 1A2, and 1A3), both ways together resulting in enhanced ability of self-renewal, survival, proliferation, and drug-resistance

As the previous results have confirmed that there are only nine potential ALDH^a^ isoforms in human cells, we wonder whether there are unique expression patterns of the ALDH^a^ isoforms for different cancer types. Therefore, we re-analyzed the data retrieved from CCLE for the selected cancer types. Interestingly, we indeed found that different cancer types exhibit different expression patterns of ALDH^a^ isoforms (Fig. 8). For example, liver cancer and kidney cancer show high level of ALDH1A1 (Fig. 8d, f), whereas breast cancer (Fig. 8a), ovary cancer (Fig. 8c), skin cancer (Fig. 8e), pancreatic cancer (Fig. 8g), and esophagus cancer (Fig. 8h) express higher level of ALDH1A3. On the other hand, lung cancer (Fig. 8b) exhibits a mixed feature of ALDH1A1 and ALDH1A3. We also observed that ALDH2 is expressed at high level by almost all seven analyzed cancer types except for skin cancer (Fig. 8a-h), whereas the expressions of ALDH3A1 and ALDH3A2 are higher in lung, pancreatic, and esophagus cancer (Fig. 8b, g–h). However, no cancer-type specific expression preference of ALDH1A2, ALDH1B1, ALDH3B1, and ALDH5A1 has been observed. ALDH1A2 is expressed at very low level in all types of cancer, whereas ALDH1B1, ALDH3B1, and ALDH5A1 are expressed at medium level universally (Fig. 8a–h), implying a ubiquitous role of these isoforms. As ALDEFLUOR assay has been applied in characterizing CSCs in many types of cancer, our results may imply that CSCs in different cancers utilize unique ALDH^a^ isoform or combinations in maintaining their stem-like characteristics.

Taken together, our results demonstrate that there are nine ALDH isoforms that potentially contribute to ALDEFLUOR assay in characterizing cancer stem cells (Fig. 8i) and reveal a cancer-type specific expression pattern of these ALDH^a^ isoforms (Fig. 8a–h). ALDH^a^ isoforms are involved in CSC regulation via two possible mechanisms (Fig. 8i). Firstly, ALDH^a^ isoforms may directly detoxify aldehydes (R-CHO) by converting them to their corresponding acids (R-COOH), the former of which is detrimental to DNA, mitochondrion, and other cellular components, and this protection role is vital to the self-renewal, survival, and proliferation of CSCs. ALDH^a^ isoform may also protect CSCs from drugs whose active metabolites contain aldehyde group (Hilton 1984). Secondly, ALDH1 family members (ALDH1A1, ALDH1A2, and ALDH1A3) are able to convert retinal to retinoic acid (RA), which binds to retinoic acid receptor (RAR)/estrogen receptor α (ERα) complex and activates the transcription of target genes such as c-Myc and Cyclin-D (Tomita et al. 2016), subsequently resulting in enhanced self-renewal, survival, and proliferation of CSCs.

## Discussions

Aldehyde dehydrogenases are a superfamily of genes with preference to different substrates and exert a variety of functions in cells (Marchitti et al. 2008), including detoxification of the detrimental aldehydes derived from endogenous metabolism or absorbed from environment. The ALDEFLUOR assay was initially developed to detect activity of ALDH1A1 in leukemia and subsequently applied to many other cancer types, such as breast (Ginestier et al. 2007) and prostate (van den Hoogen et al. 2010). Due to this historical reason, the ALDH activity detected by ALDEFLUOR assay has long been solely owed to ALDH1A1, although 19 ALDH isoforms have been identified in the human genome and some of them have recently been demonstrated to catalyze the ALDEFLUOR assay. Till now, it is still a partially answered open question that which of the 19 ALDH isoforms are contributors in ALDEFLUOR assay, forcing researchers to subconsciously assume that all ALDH isoforms are equally active in ALDEFLUOR assay. From our results, we could conclude that only nine ALDH isoforms are active in ALDEFLUOR assay (ALDH^a^ isoforms). Besides the classical ALDH1A1 and some other previously reported ALDH isoforms, we have also identified several novel active ALDH isoforms that have not been reported by others to be involved in ALDEFLUOR assay, which would provide new research focus in the field of ALDH^+^ marked cancer stem cells and may possibly lead to discovery of novel therapeutic targets. These results further lead us to another open question: which of these active isoforms are underlying players in the CSC regulation.

However, we cannot rule out the possibility that the so-called ALDH^n^ isoforms, showing no activities in ALDEFLUOR assay, may contribute to cancer stem cell regulation in a non-enzymatic manner or by catalyzing substrates that are different to BAAA in structure. For example, ALDH7A1 has been reported to regulate the cancer stem cell self-renewal in colorectal, prostate cancer, and glioblastoma as a downstream effector of Wnt pathway (Prabhu et al. 2017). Besides, as we have only tested the ALDH isoforms in limited cell lines, it is also possible that some of the ALDH^n^ isoforms may contribute the ALDH activity in ALDEFLUOR assay in other cancer types with unique intracellular context.

Additionally, inhibition of ALDH activity has been applied for various research or therapeutic aims, as there are 19 ALDH isoforms in human genome and they exert different functions in both physiological and pathological conditions. As one of these inhibitors, DEAB used to be identified as an ALDH1A1-specific inhibitor and used in the ALDEFLUOR assay to inhibit ALDH activity so as to provide a negative control. However, C.A. Morgan et al. previously demonstrated DEAB to be an inhibitor to several ALDH isoforms based on different mechanisms in vitro (Morgan et al. 2015). Our results also confirmed part of their results that DEAB is not only an inhibitor to ALDH1A1 but also to several other ALDH^a^ isoforms in ALDEFLUOR assay. What is more interesting in our results is that DEAB is poor or even not an inhibitor to the ALDH3 family (ALDH3A1, ALDH3A2, and ALDH3B1) and ALDH5A1, which demands the development of specific inhibitors to these isoforms.

Our analysis of the expression patterns of ALDH^a^ isoforms revealed cancer-type specific expression patterns. It is noteworthy that some isoforms, such as ALDH2, ALDH3A2, ALDH3B1, and ALDH5A1, are expressed at similar levels across cancer types. However, their role in ALDEFLUOR assay in these cancer types may vary due to different cellular context, e.g., post-transcriptional regulation and post-translational modification pressure. In fact, we have noticed that not all cells are ALDH-positive for the ALDH^a^ isoforms, although we established stable overexpressing cell lines and the ALDH^+^ proportion are significantly increased (Figs. 2b, 3b and 4b). To determine whether this was a result of differential expression level in two populations, we detected the expression level in sorted ALDH^−^ and ALDH^+^ populations and found that the two populations expressed similar amount of ALDH mRNA and protein (unpublished data). This result strongly implies that the activity of ALDH isoforms is partially regulated at post-translational level, where some key modifications may influence the activity of ALDH protein. In fact, several papers have recently reported that the activity of ALDH1A1 is regulated by acetylation (Zhao et al. 2014) and phosphorylation (Wang et al. 2017). As we have identified more other ALDH isoforms potentially involved in CSC identification by ALDEFLUOR assay, much work remains to be done to unravel the complex post-translational modification (PTM) regulation of ALDH activity for other ALDH^a^ isoforms in CSCs.

In summary, we have systematically screened the 19 ALDH isoforms in the human genome and identified nine isoforms that are able to catalyze ALDEFLUOR assay and thus are potentially contributors in characterizing cancer stem cells. Our work also supports previous studies that DEAB, the inhibitor used in ALDEFLUOR assay, is not specific to ALDH1A1 but to a broad spectrum of ALDH isoforms and we find that DEAB is a poor or even not an inhibitor to ALDH3 family and ALDH5A1. Furthermore, our results strongly imply that the activity of all ALDH^a^ isoforms may be probably regulated at post-transcriptional level, as the case of ALDH1A1 whose activity has been reported to be regulated by acetylation (Zhao et al. 2014) and phosphorylation (Wang et al. 2017). Our analysis of the expression of ALDH isoforms also reveals cancer-type specific expression patterns, which may imply that different cancer types utilize different ALDH^a^ isoforms to exert their ALDEFLUOR activity. This phenomenon would further promote the demands of developing ALDH isoform specific inhibitors to target CSCs in different cancers.

## Electronic supplementary material

ESM 1 Supplementary data for this study is available in Cell Biology and Toxicology online. Materials mentioned in this manuscript are available from the corresponding author upon a reasonable request. (DOCX 2519.04 kb)

## Abbreviations

ALDH: Aldehyde dehydrogenase
ALDH^a^: Active ALDH isoform
ALDH^n^: Non-active ALDH isoform
BAAA: BODIPY-aminoacetaldehyde
BAA: BODIPY-aminoacetate
CCLE: Broad Institute Cancer Cell Line Encyclopedia
CSC: Cancer stem cell
DEAB: *N*,*N*-Diethylaminobenzaldehyde
GAPDH: Glyceraldehyde-3-phosphate dehydrogenase
TIC: Tumor-initiating cell
Gag/pol: Gag-Pol polyprotein
VSVG: Vesicular stomatitis virus (VSV) G protein
