# Supplementary material for: Identification of cancer-type specific expression patterns for active aldehyde dehydrogenase (ALDH) isoforms in ALDEFLUOR assay
Source: Cell Biol Toxicol. 2018 Sep 15;35(2):161–77. doi: 10.1007/s10565-018-9444-y (PMC6424948; doi:10.1007/s10565-018-9444-y)
Supplement: Supplementary file 1 — Supplementary data for this study is available in Cell Biology and Toxicology online. Materials mentioned in this manuscript are available from the corresponding author upon a reasonable request. (DOCX 2519.04 kb) [file 10565_2018_9444_MOESM1_ESM.docx]

**Additional file**

**Supplementary Figures and Tables:**

**
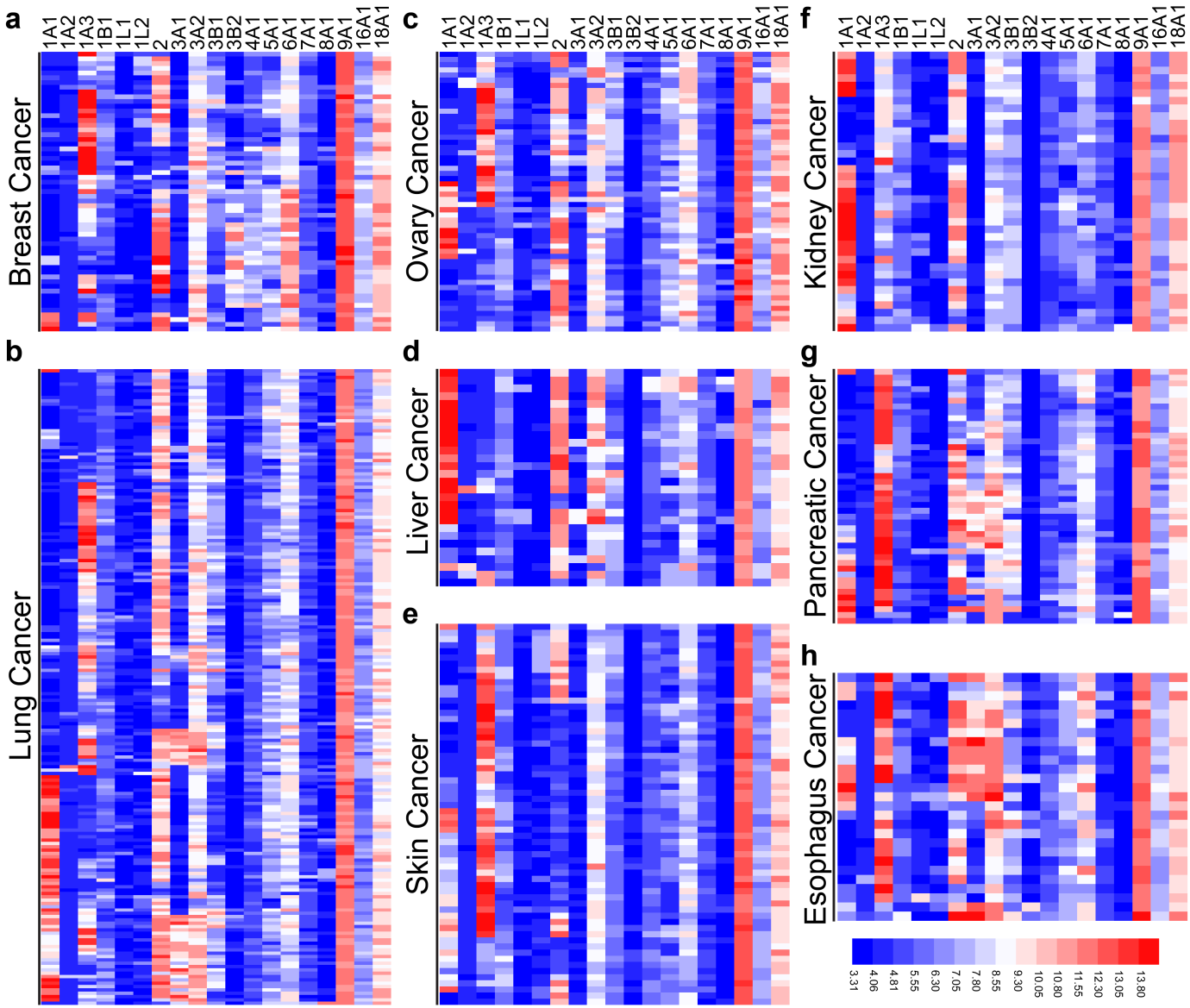
**

**Figure S1. ALDH isoforms are expressed in cancer-type specific patterns.** RNA expression data for all 19 ALDH isoforms was collected from CCLE and illustrated as heatmap by HemI ([Deng et al., 2014](#_ENREF_1)),clustered with hierarchical maximum linkage. Data was retrieved and analyzed for breast cancer (**a**), lung cancer (**b**), ovary cancer (**c**), liver cancer (**d**), skin cancer (**e**), kidney cancer (**f**), pancreatic cancer (**g**), and esophagus cancer (**h**). Heat map was clustered with hierarchical average linkage using the HemI software.

**Table S1**

**Primers used to clone ALDH isoforms.**

| **Genes*** | **Forward primer (5’ to 3’)** | **Reverse primer (5’ to 3’)** |
| --- | --- | --- |
| FLAG-ALDH1A1 | ACGATGATGACAAAGAATTCATGTCATCCTCAGGCACG | TCCTTCGAACTAGTACGCGTTTATGAGTTCTTCTGAGAGAT |
| FLAG-ALDH1A2 | ACGATGATGACAAAGAATTCATGACTTCCAGCAAGATAG | TCCTTCGAACTAGTACGCGTTTAGGAGTTCTTCTGGGGGAT |
| FLAG-ALDH1A3 | ACGATGATGACAAAGAATTCATGGCCACCGCTAACGG | TCCTTCGAACTAGTACGCGTTCAGGGGTTCTTGTCGCCAAG |
| ALDH1B1-FLAG | GTCGTGAGGAATTGGGATCCGCCACCATGCTGCGCTTCCTGGCA | GAGCCTCCACCCCCACGCGTCGAGTTCTTCTGAGGAAC |
| ALDH1L1-FALG | GTCGTGAGGAATTGGGATCCGCCACCATGGCAGGTCCTTCCAAC | GAGCCTCCACCCCCACGCGTGTATTCGAAGGTCACTGT |
| ALDH1L2-FLAG | GTCGTGAGGAATTGGGATCCGCCACCATGCTGCGGCGGGGC | GAGCCTCCACCCCCACGCGTATATTCCAGTGTCACCG |
| ALDH2-  FLAG | GTCGTGAGGAATTGGGATCCGCCACCATGTTGCGCGCTGCCGC | AGAGCCTCCACCCCCACGCGTTGAGTTCTTCTGAGGCACT |
| FLAG-ALDH3A1 | ACGATGATGACAAAGAATTCATGAGCAAGATCAGCG | TCCTTCGAACTAGTACGCGTTCAGTGCTGGGTCATCT |
| FALG-ALDH3A2 | ACGATGATGACAAAGAATTCATGGAGCTCGAAGTCCG | TCCTTCGAACTAGTACGCGTTCATCTCTGCTTACTGGAC |
| FLAG-ALDH3B1 | GACGATGATGACAAAGAATTCATGGACCCCCTTGGG | TCCTTCGAACTAGTACGCGTTCAGAGCAGTGTGCAGCT |
| FALG-ALDH3B2 | GACGATGATGACAAAGAATTCATGAAGGATGAACCACGGT | TCCTTCGAACTAGTACGCGTTCACAGGAGGGTGCAGCT |
| ALDH4A1-FLAG | GTCGTGAGGAATTGGGATCCGCCACCATGCTGCTGCCGGCGC | GAGCCTCCACCCCCACGCGTCTGCATGTACGCGTAG |
| ALDH5A1-FLAG | GTCGTGAGGAATTGGGATCCGCCACCATGGCGACCTGCATTT | GAGCCTCCACCCCCACGCGTCAAGCCCCCGTAACACAC |
| ALDH6A1-FLAG | GTCGTGAGGAATTGGGATCCGCCACCATGGCGGCGCTATTGG | GAGCCTCCACCCCCACGCGTACGGCCCATGGTAGGCAT |
| FLAG-ALDH7A1 | ACGATGATGACAAAGAATTCATGTCCACTCTCCTCAT | TCCTTCGAACTAGTACGCGTTTACTGAAACTTGATTCCTT |
| FLAG-ALDH8A1 | ACGATGATGACAAAGAATTCATGGCTGGAACAAACGCAC | TCCTTCGAACTAGTACGCGTTCAGTGTTTAACGGTGATGGT |
| FLAG-ALDH9A1 | ACGATGATGACAAAGAATTCATGTTTCTCCGAGCAGG | TCCTTCGAACTAGTACGCGTTCAAAAAGCAGATTCCACAT |
| FLAG-ALDH16A1 | GACGATGATGACAAAGAATTCATGGCTGCGACGCGTG | TCCTTCGAACTAGTACGCGTTCAGTCCCCCATAGGCAG |
| ALDH18A1-FLAG | GTCGTGAGGAATTGGGATCCGCCACCATGTTGAGTCAAGTTTACCGCT | GAGCCTCCACCCCCACGCGTGTTGGTGTTTCTCTGAGGAAT |

*Note: The FLAG tag is shown together with ALDH isoforms to indicate the N-erminal or C-terminal fusion pattern.

**Table S2**

**Primers for mutation of the activity center of ALDH^a^ isoforms.**

| **Genes** | **Forward primer (5’ to 3’)** | **Reverse primer (5’ to 3’)** |
| --- | --- | --- |
| ALDH1A1-C303A | ACCACCAGGGCCAGTGTGCTATAGCCGCATCCAGGAT | ACACTGGCCCTGGTGGT |
| ALDH1A2-C322A | TTCAATCAAGGTCAGTGCGCCACTGCAGGCTCTCGC | GCACTGACCTTGATTGAA |
| ALDH1A3-C314A | CAACCAAGGCCAGTGTGCCACGGCAGCCTCCAG | ACACTGGCCTTGGTTGA |
| ALDH1B1-C319A | AACATGGGCCAGTGCGCCTGTGCTGGCTCCCGG | GCACTGGCCCATGTT |
| ALDH2-C301A | AACCAGGGCCAGGCCTGCTGTGCCGGCTCCCGG | GCACTGGCCCTGGTT |
| ALDH3A1-C244A | GAACAGTGGCCAGACCGCCGTGGCCCCTGACTACAT | GGTCTGGCCACTGTTC |
| ALDH3A2-C241A | GAATTGTGGCCAAACCGCCATTGCACCCGACTATATTCT | GGTTTGGCCACAATTCATG |
| ALDH3B1-C244A | AACGCCGGCCAGACCGCCGTGGCCCCCGACTACG | GGTCTGGCCGGCGTTG |
| ALDH5A1-C353A | GAACACTGGACAGACTGCCGTTTGCTCAAACCAATTCTTG | AGTCTGTCCAGTGTTCCT |

**Table S3**

**Primers used in quantitative real-time PCR (qRT-PCR).**

| **Genes** | **Forward primer (5’ to 3’)** | **Reverse primer (5’ to 3’)** |
| --- | --- | --- |
| ALDH1A1 | TGTTAGCTGATGCCGACTTG | TTCTTAGCCCGCTCAACACT |
| ALDH1A2 | GGGTGTGTTCTTCAATCAAGGT | TGGTGGGGTCAAAGGGACT |
| ALDH1A3 | TCTCGACAAAGCCCTGAAGT | TATTCGGCCAAAGCGTATTC |
| ALDH1B1 | CTGGAGCTGGGTGGTAAGAG | CTTTCTCCACGGTTCTCTCG |
| ALDH1L1 | TCCAGACCTTCCGCTACTTTG | CAGGGGATAGTTCCAGGGGAT |
| ALDH1L2 | GCCTGGTCTCGTTACCAAAA | GCCACTTTCACCTCTTCAGC |
| ALDH2 | CCTCACCGCCCTCTATGTG | CGGCCAATCTCAGTGGAGC |
| ALDH3A1 | GCAGACCTGCACAAGAATGA | TGTAGAGCTCGTCCTGCTGA |
| ALDH3A2 | TGCACTTCACGCTCAACTCT | GACTGGCTGTTGGGAGGATA |
| ALDH3B1 | ACAAGTCAGCCTTCGAGTCGG | AGCACCACACAGTTCCCTGC |
| ALDH3B2 | ACAGAGAAGGTCCTGGCTGA | CATGACAATCTTGCCCACAC |
| ALDH4A1 | GTACGGTGGCCAGAAGTGTT | TCTTGATACGGGCAAAGGAC |
| ALDH5A1 | ACCAATTCTTGGTGCAAAGG | GTTGGTGTCGTTTTCCACCT |
| ALDH6A1 | GGCTCTTTCAACAGCAGTCC | ATGGAAGCTCCCTCCTTTGT |
| ALDH7A1 | CGAGCCAATAGCAAGAGTCC | CTTCACCCACACCTTCCACT |
| ALDH8A1 | TGGTGAGCATAGGTGCTCTG | GTTATCACCGTGGGAAGCAT |
| ALDH9A1 | CACTCATCAACCGACCACAC | GGACATAACAGGCCCAAAGA |
| ALDH16A1 | GGGCTGTTCGAGAGGTTCG | CAGGGCAAATCCTCCACATCA |
| ALDH18A1 | CTGAGTATGGGGACCTGGAA | GCGGTAACCATCAGAAAAGC |
| GAPDH | ATGGGGAAGGTGAAGGTCG | GGGGTCATTGATGGCAACAATA |

**Table S4**

**Primers used to constructe shRNA vectors.**

| **shRNA clone** | **Forward primer (5’ to 3’)** | **Primer length**  **(nucleotides)** |
| --- | --- | --- |
|  | **Reverse primer (5’ to 3’)** |  |
| **Scramble shRNA** | ctcgagTTGGTGCTCTTCATCTTGTTGTTTTTGAATTCTCGACCTCGAG | 49 |
|  | ACCAActcgagTTGGTGCTCTTCATCTTGTTGCGGTGTTTCGTCCTTTCC | 50 |
| **ALDH1A1-sh1** | ctcgagAATTCCAAGGAATGATTTGGCTTTTTGAATTCTCGACCTCGAG | 49 |
|  | GAATTctcgagAATTCCAAGGAATGATTTGGCGGTGTTTCGTCCTTTCC | 49 |
| **ALDH1A1-sh2** | ctcgagATCTCTTTCGATTAAATCAGCTTTTTGAATTCTCGACCTCGAG | 49 |
|  | GAGATctcgagATCTCTTTCGATTAAATCAGCGGTGTTTCGTCCTTTCC | 49 |
| **ALDH1A1-sh3** | ctcgagAAGGATATACTTCTTAGCCCGTTTTTGAATTCTCGACCTCGAG | 49 |
|  | TCCTTctcgagAAGGATATACTTCTTAGCCCGCGGTGTTTCGTCCTTTCC | 50 |
| **ALDH1A1-sh4** | ctcgagTTGACCTCTGTATATTCATGGTTTTTGAATTCTCGACCTCGAG | 49 |
|  | GTCAActcgagTTGACCTCTGTATATTCATGGCGGTGTTTCGTCCTTTCC | 50 |
| **ALDH1A1-sh5** | ctcgagTATTGAATCTTCAAATCGGTGTTTTTGAATTCTCGACCTCGAG | 49 |
|  | CAATActcgagTATTGAATCTTCAAATCGGTGCGGTGTTTCGTCCTTTCC | 50 |
| **ALDH1A3-sh1** | ctcgagATCTGAGGGTTCTAATACAGCTTTTTGAATTCTCGACCTCGAG | 49 |
|  | CAGATctcgagATCTGAGGGTTCTAATACAGCGGTGTTTCGTCCTTTCC | 49 |
| **ALDH1A3-sh2** | ctcgagTTGAACTTCAGTATTGGTTGCTTTTTGAATTCTCGACCTCGAG | 49 |
|  | TTCAActcgagTTGAACTTCAGTATTGGTTGCGGTGTTTCGTCCTTTCC | 49 |
| **ALDH1A3-sh3** | ctcgagATACGACGTTGTCATCTGTGGTTTTTGAATTCTCGACCTCGAG | 49 |
|  | CGTATctcgagATACGACGTTGTCATCTGTGGCGGTGTTTCGTCCTTTCC | 50 |
| **ALDH1A3-sh4** | ctcgagTTTCACTTCTGTGTATTCGGCTTTTTGAATTCTCGACCTCGAG | 49 |
|  | TGAAActcgagTTTCACTTCTGTGTATTCGGCGGTGTTTCGTCCTTTCC | 49 |
| **ALDH1A3-sh5** | ctcgagAAACTCAGAGTAGACCTGCTCTTTTTGAATTCTCGACCTCGAG | 49 |
|  | AGTTTctcgagAAACTCAGAGTAGACCTGCTCGGTGTTTCGTCCTTTCC | 49 |
| **ALDH2-sh1** | ctcgagTTTCCCGTGGTACTTATCAGCTTTTTGAATTCTCGACCTCGAG | 49 |
|  | GGAAActcgagTTTCCCGTGGTACTTATCAGCCGGTGTTTCGTCCTTTCC | 50 |
| **ALDH2-sh2** | ctcgagATCTGACATGATGATGTTGGGTTTTTGAATTCTCGACCTCGAG | 49 |
|  | CAGATctcgagATCTGACATGATGATGTTGGGCGGTGTTTCGTCCTTTCC | 50 |
| **ALDH2-sh3** | ctcgagTTCACTTCAGTGTATGCCTGCTTTTTGAATTCTCGACCTCGAG | 49 |
|  | GTGAActcgagTTCACTTCAGTGTATGCCTGCGGTGTTTCGTCCTTTCC | 49 |
| **ALDH2-sh4** | ctcgagAAATTCCACGGAATGATCTGCTTTTTGAATTCTCGACCTCGAG | 49 |
|  | AATTTctcgagAAATTCCACGGAATGATCTGCGGTGTTTCGTCCTTTCC | 49 |
| **ALDH2-sh5** | ctcgagAAACTCATCATAGATGTCCTCTTTTTGAATTCTCGACCTCGAG | 49 |
|  | AGTTTctcgagAAACTCATCATAGATGTCCTCGGTGTTTCGTCCTTTCC | 49 |
| **ALDH3A2-sh1** | ctcgagTTCACAGAGAATATAGTCGGGTTTTTGAATTCTCGACCTCGAG | 49 |
|  | GTGAActcgagTTCACAGAGAATATAGTCGGGCGGTGTTTCGTCCTTTCC | 50 |
| **ALDH3A2-sh2** | ctcgagTTGATGAGCTTATGGTTATGCTTTTTGAATTCTCGACCTCGAG | 49 |
|  | ATCAActcgagTTGATGAGCTTATGGTTATGCGGTGTTTCGTCCTTTCC | 49 |
| **ALDH3A2-sh3** | ctcgagAATTCACTCTTGCACAGGTCGTTTTTGAATTCTCGACCTCGAG | 49 |
|  | GAATTctcgagAATTCACTCTTGCACAGGTCGCGGTGTTTCGTCCTTTCC | 50 |
| **ALDH3A2-sh4** | ctcgagTATAATCACAGCATTTCCTGCTTTTTGAATTCTCGACCTCGAG | 49 |
|  | TTATActcgagTATAATCACAGCATTTCCTGCGGTGTTTCGTCCTTTCC | 49 |
| **ALDH3A2-sh5** | ctcgagAAATACATAAAGAGCCAGAGGTTTTTGAATTCTCGACCTCGAG | 49 |
|  | TATTTctcgagAAATACATAAAGAGCCAGAGGCGGTGTTTCGTCCTTTCC | 50 |
| **ALDH3B1-sh1** | ctcgagTTCTTGCTAATCTCCGATGGCTTTTTGAATTCTCGACCTCGAG | 49 |
|  | AAGAActcgagTTCTTGCTAATCTCCGATGGCGGTGTTTCGTCCTTTCC | 49 |
| **ALDH3B1-sh2** | ctcgagTTCTCGACGTTCTTGCTAATCTTTTTGAATTCTCGACCTCGAG | 49 |
|  | GAGAActcgagTTCTCGACGTTCTTGCTAATCCGGTGTTTCGTCCTTTCC | 50 |
| **ALDH3B1-sh3** | ctcgagACGCTCGTCCTTCATCCAGGCTTTTTGAATTCTCGACCTCGAG | 49 |
|  | AGCGTctcgagACGCTCGTCCTTCATCCAGGCGGTGTTTCGTCCTTTCC | 49 |
| **ALDH3B1-sh4** | ctcgagAACCTCAGACACCTCCGACTCTTTTTGAATTCTCGACCTCGAG | 49 |
|  | AGGTTctcgagAACCTCAGACACCTCCGACTCGGTGTTTCGTCCTTTCC | 49 |
| **ALDH3B1-sh5** | ctcgagGGGTCATGTGCATGAAGCCGTTTTTTGAATTCTCGACCTCGAG | 49 |
|  | CCTTTctcgagGGGTCATGTGCATGAAGCCGTGGTGTTTCGTCCTTTCC | 49 |

**Note:** Forward and reverse primers used for construction of shRNA vectors targeting ALDH1A1, ALDH1A3, ALDH2, ALDH3A2 and ALDH3B1 were listed, with guide (antisense) sequences highlighted in red and loop sequences in lowercase.

**Reference:**

Deng, W., Wang, Y., Liu, Z., Cheng, H., and Xue, Y. (2014). HemI: a toolkit for illustrating heatmaps. PloS one *9*, e111988.
